# Subjective symptoms and serum methadone concentrations: what should guide dose adjustments in methadone maintenance treatment? A naturalistic cohort study from Norway

**DOI:** 10.1186/s13011-021-00367-w

**Published:** 2021-05-03

**Authors:** Fatemeh Chalabianloo, Lars Thore Fadnes, Gudrun Høiseth, Christian Ohldieck, Jørn Henrik Vold, Christer Aas, Else-Marie Løberg, Kjell Arne Johansson, Jørgen G. Bramness

**Affiliations:** 1 Department of Addiction Medicine, Haukeland University Hospital, Bergen, Norway; 2 Department of Global Public Health and Primary Care, University of Bergen, Bergen, Norway; 3 Department of Forensic Medicine, Oslo University Hospital, Oslo, Norway; 4 Center for Psychopharmacology, Diakonhjemmet Hospital, Oslo, Norway; 5 Norwegian Center for Addiction Research, University of Oslo, Oslo, Norway; 6 Institute of Clinical Psychology, University of Bergen, Bergen, Norway; 7 Division of Psychiatry, Haukeland University Hospital, Bergen, Norway; 8 Institute of Clinical Medicine, UiT – Norway’s Arctic University, Tromsø, Norway; 9 Department of Alcohol, Tobacco and Drugs, Norwegian Institute of Public Health, Oslo, Norway

**Keywords:** Methadone maintenance treatment, Serum concentrations, Subjective opioid withdrawal symptoms, Adverse effects, Substance use, Opioid agonist treatment

## Abstract

**Background:**

There is little evidence-based guidance on how to optimize methadone dosages among patients with opioid addiction undergoing methadone maintenance treatment (MMT). This study aims to investigate whether self-perceived opioid withdrawal symptoms, adverse effects, and self-reported substance use in patients on MMT are related to serum methadone concentrations and the role that these variables could play in clinical decisions on dose adjustments.

**Methods:**

This naturalistic prospective cohort study included clinical and laboratory measurements from 83 patients undergoing MMT in outpatient clinics in Bergen, Norway, from May 2017 to January 2020. Information on age, gender, methadone daily doses and serum concentrations, subjective opioid withdrawal symptoms using 16 items Subjective Opioid Withdrawal Scale (SOWS) questionnaire, self-reported adverse effects, and substance use was obtained. Linear mixed modelling was used for analyzing the data.

**Results:**

The mean age of the participants was 45 years, and 33% were women. Almost half reported mild to moderate subjective opioid withdrawal symptoms, and all had experienced at least one subjective adverse effect. The use of at least one substance was reported by 88% of the participants. Serum concentration-to-dose ratios were lower among those who had reported subjective opioid withdrawal symptoms (*p)* = 0.039). The total SOWS score (*p* < 0.001); the specific subjective withdrawal symptoms of anxiety (*p* = 0.004), bone and muscle aches (*p* = 0.003), restlessness (*p* = 0.017), and (slightly) shaking (*p* = 0.046), also use of heroin (*p* = 0.015) and alcohol (*p* = 0.011) were associated with lower methadone concentrations. Cannabis use was slightly related to higher methadone concentrations (*p* = 0.049).

**Conclusions:**

The findings suggest that the patient’s self-perceived symptoms and current clinical condition are related to the serum concentrations of methadone. This interpretation supports dose adjustments based on patient-reported symptoms. In some aberrant cases, measurement of serum concentrations together with other individual assessments may be considered to support the clinical decision.

## Background

Methadone maintenance treatment (MMT) is an evidence-based medical intervention that reduces illicit opioid use and risk of overdose [1, 2] and mortality [3, 4] among opioid-dependent individuals. Understanding factors that may influence treatment satisfaction and continuity – and accordingly preventing a relapse to illicit opioid use and the subsequent risk of overdose and death – is crucial. Such factors may be opioid withdrawal symptoms and adverse effects related to inappropriate methadone dosages. Thus, balancing an efficacious dose to achieve the desired therapeutic effect against a dose that is either too low, leading to withdrawal symptoms and relapse to illicit opioid use or too high, causing adverse effects and toxicity, is important in clinical practice.

Individualized dose optimization using daily doses of between 60 and 120 mg for most patients appears to be related to increased retention in treatment and reduced illicit opioid use as the most common measures of MMT efficacy [5]. However interindividual variations in methadone dose requirements should be kept in mind [6–8]. Furthermore, some researchers have shown that factors other than the dose – such as the patient’s expectations and medication preferences, as well as the patient’s total physical and mental health condition or improvements in psychosocial functioning – may influence treatment satisfaction [9, 10]. These findings add to the complexity of the issue challenging clinicians regarding how to cope with suboptimal treatment outcomes: should the dose be adjusted, or should other problems instead be addressed?

One can predict the optimal methadone maintenance dose using various factors based on continuous clinical evaluations [11–13]. Although previous findings suggest an association between the dose and clinical symptoms, the relationships between serum methadone concentrations and treatment effects are still not fully understood [14]. Due to the large interindividual variation in methadone pharmacokinetics, individual serum concentrations after a given dose will vary substantially [12, 14–16]. The optimal dose at steady state thus is hard to predict [12]. Accordingly, a clinically oriented approach rather than an approach based on serum levels has been suggested for optimizing the methadone daily dosage for individual patients [11, 12, 17].

Limited studies [18, 19], however, have suggested a direct association between the serum methadone concentration and retention in treatment. A few clinical studies [20, 21] have shown that when the serum concentration is too low to inhibit objective withdrawal symptoms, patients relapse to substance use and drop out of MMT. Other studies [11, 22, 23] have indicated that higher concentrations are more likely to reduce opioid craving. Finally, a rapid decline in the trough concentration is related to clinically important responses, notably objective withdrawal symptoms [24]. However, there is limited knowledge on the specific subjective symptoms reflecting the serum concentrations of methadone, which clinicians must recognize to guide proper dose adjustments in clinical practice.

The aim of this study is to investigate whether self-perceived opioid withdrawal symptoms, adverse effects, and substance use in patients on MMT are related to serum methadone concentrations and the role that these variables should play in clinical decisions on dose adjustments.

## Methods

### Design, setting, and data sources

This naturalistic prospective cohort study was conducted at the Department of Addiction Medicine at Haukeland University Hospital in Bergen, Norway, from May 2017 to January 2020. The department is responsible for the treatment and follow-up of more than 1000 patients with opioid dependence receiving opioid agonist treatment (OAT). Almost 40% of these patients receive MMT, while the rest mainly receive buprenorphine-based treatment. All pharmacological interventions are integrated with psychosocial treatment and are provided in multidisciplinary outpatient clinics. The patients are followed-up via directly observed treatment (DOT) and consultations, and take-home doses frequencies are based on the individual treatment courses. All clinical measurements and laboratory data are recorded prospectively in the hospital journal system, as well as in a recently established health registry database for patients undergoing OAT in Bergen. In addition to incorporating individual health data, the database includes relevant information based on clinical surveys and research records.

In the present study, we included information on age, gender, daily methadone doses, serum methadone concentrations (as the independent variable), subjective opioid withdrawal symptoms (as the primary outcome), some common subjective opioid adverse effects and self-reported illicit drug use (as the secondary outcomes). We also included information about the time since last dose intake, time of blood sampling, time when symptoms were recorded, numbers of days with DOT per week, and duration of OAT.

### Participants

One hundred and ninety-nine patients consented to participate in the study and started the primary surveys through in-person clinical interviews by a research nurse. At the end of the study, 83 patients had completed the surveys according to the study protocol, with the time difference between the clinical assessments and laboratory measurements being < 14 days (Mean = 2, SD = 3), and were included in the study.

### Assessments of subjective symptoms

As part of the clinical assessments and based on the study protocol, the participants were initially asked whether they were experiencing opioid withdrawal symptoms. Those who confirmed the presence of withdrawal symptoms were interviewed by a research nurse using the validated standard questionnaire, the Subjective Opioid Withdrawal Scale (SOWS) [25], which covers 16 self-perceived symptoms: anxiety, yawning, perspiring, tearing, runny nose, goosebumps, shaking, hot flushes, cold flushes, bone and muscle aches, restlessness, nausea, vomiting, muscle twitches, stomach cramps, and feeling like using. Respondents rated the intensity of symptoms on a five-point scale from 0 (not at all) to 4 (extreme); possible scores range from 0 to 64 (1–10 = mild withdrawal, 11–20 = moderate withdrawal, 21–30 = severe withdrawal).

In addition, all participants were asked six questions on some of the most common subjective adverse effects attributed to MMT, including euphoria, perspiring, nausea, concentration difficulties, feeling “brain fog,” and reduced sexual desire; these symptoms were rated in the same way as for the withdrawal symptoms. The selection of the adverse effects was based on the authors clinical experiences, previously published peer-collected data on this population [10], and the most common reported side effects for methadone [26, 27] and opioids in general [28]. Perspiring and nausea were defined as adverse effects or withdrawal symptoms based on how each participant perceived them. In addition, one open-ended question asked about other possible symptoms when these were self-perceived to be related to MMT.

### Substance use

Self-reported use of illicit drugs – including heroin or other opioids, amphetamines (amphetamine and/or methamphetamine), benzodiazepines, and cannabis – as well as alcohol, and frequencies of use (categorized as no use at all, or frequent use including either several times a month, weekly, or daily use) during the last month were recorded for the participants.

### Measurements of serum methadone concentrations

Blood samples were drawn from the participants at the OAT clinics according to the study protocol on average 21 (SD = 8) hours after the last dose intake, and no changes in dosages were made during the week prior to sampling. Analysis of methadone was performed by the same analytical method using the same laboratory instruments at the Department of Medical Biochemistry and Clinical Pharmacology, Haukeland University Hospital, Bergen. During the development phase of the method and in routine use, methadone concentrations were measured in nmol/L. The conversion factor from nmol/L to ng/mL for methadone is 0.310.

### Statistical analyses

Basic descriptive statistical analysis of the data was performed using Stata/SE 16.0 (StataCorp, TX, USA). Continuous variables were presented as means with standard deviations (SD), as well as ranges when needed. Comparisons of study variables between the participants with and without reported subjective opioid withdrawal symptoms were performed using Mann–Whitney tests for continuous variables and chi-square tests for categorical variables. The exact *p*-values were reported, and values < 0.05 were considered statistically significant. To avoid type-II statistical errors by overlooking important variables due to the study’s naturalistic design, we also included variables with a *p*-value < 0.10 in the adjusted regression analyses.

Linear mixed model (LMM) analysis was applied to investigate possible associations according to the aim of the study. We included in the main analyses all 16 SOWS items, the 6 subjective adverse effects, and self-reported substance use during the month prior to interviews as dependent variables. The responses to the open-ended question were excluded from the statistical analyses due to scant applicable data. All these variables were included one by one in the unadjusted statistical analyses. Then, adjusted LMM analyses for the specific variables showing statistically significant associations with the serum methadone concentration were undertaken. The results obtained in the main analyses were adjusted for age, gender, and the absolute time difference between blood sampling and the recording of the symptoms.

## Results

### Demographic and clinical data

Table 1 shows the demographic and clinical data of the 83 study participants and comparisons between those with and without reported withdrawal symptoms as the main outcome of the study. For all participants, the mean age was 45 (SD = 9) years; 33% were women, and 54% reported mild to moderate subjective opioid withdrawal symptoms with a mean total SOWS score of 9 (SD = 12) at the time of the interviews. The mean methadone daily dose and serum concentration were 97 (SD = 24) mg and 374 (SD = 188) ng/mL, respectively. Those who had reported subjective opioid withdrawal symptoms had lower serum concentration-to-dose ratios (*p* = 0.039), and more frequently received DOT (*p* = 0.026) compared to those in the other group. All had experienced one or more subjective adverse effects, and 73 (88%) reported frequent use of at least one substance during the month prior to the surveys. There were no differences between the groups with regard to age, gender, or self-reported use of illicit substances and alcohol.

**Table 1:** Demographic and clinical data in the study participants and comparisons between the groups with and without reported subjective opioid withdrawal symptoms

	All participants	Withdrawals	No withdrawals	
	*N* = 83	*N* = 45	*N* = 38	*p-*value^a^
Gender, female/male^b^	27/56 (33/67)	15/30 (56/54)	12/26 (44/46)	0.865
Age, years^c^	45 (9, 26–66)	45 (9, 26–62)	44 (10, 26–66)	0.493
Methadone dose, mg/day^c^	97 (24, 20–170)	101 (24, 35–170)	92 (23, 20–150)	0.079
Methadone serum concentration, ng/mL^c^	374 (188, 74–1005)	347 (168, 113–1005)	405 (208, 74–998)	0.145
CDR,^d^ (ng/mL)/(mg/day)^c^	4 (2, 1–11)	3 (2, 1–11)	4 (2, 1–9)	0.039
Time since last dose, hours^c^	21 (8, 0–28)	22 (5, 1–28)	19 (10, 0–27)	0.199
Duration of opioid agonist treatment^c^	9 (5, 1–20)	9 (5, 1–18)	9 (5, 1–20)	0.922
Direct observed treatment, day/week^c^	4 (2, 1–7)	4 (2, 1–7)	3 (2, 1–6)	0.026
Self-reported substance use last month^b^	73 (88)	41 (92)	32 (83)	0.187
*Heroin*^b^	10 (12)	5 (12)	5 (13)	0.971
*Other opioids*^b^	5 (6)	3 (6)	2 (5)	0.819
*Benzodiazepines*^b^	50 (62)	29 (64)	21 (55)	0.233
*Cannabis*^b^	55 (66)	30 (65)	25 (67)	0.828
*Amphetamine*^b^	26 (31)	15 (33)	11 (30)	0.789
*Alcohol*^b^	38 (46)	22 (49)	16 (42)	0.542

^a^Significance was tested using Mann-Whitney U-test for continuous and chi-square test for categorical variables

^b^The categorical variables are presented by n (%)

^c^The continuous variables are presented as means with standard deviations (SD) and ranges

^d^Concentration-to-dose ratio

### Relationships between subjective opioid withdrawal symptoms and serum methadone concentrations

**Fig. 1:**
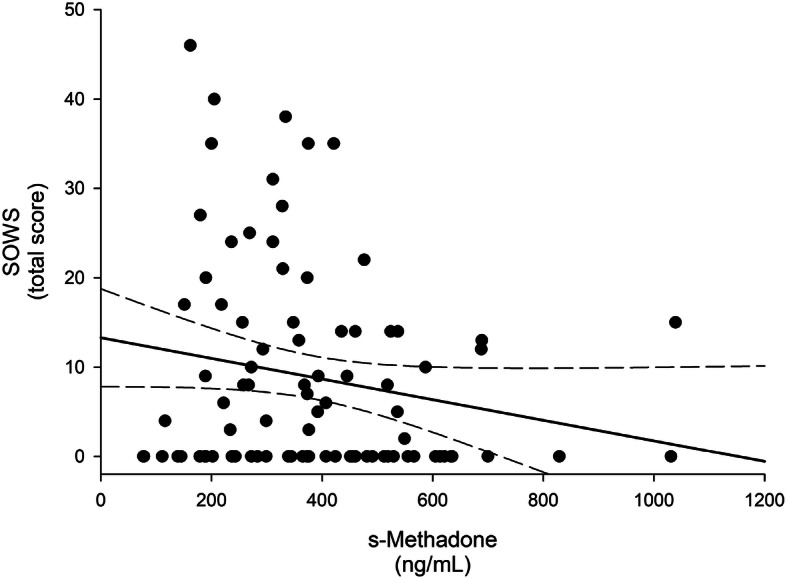
Scatter plot with regression line by recorded total SOWS^a^ scores and measured serum methadone concentrations in 83 participants. The solid and dashed lines represent the regression line and 95% confidence intervals. ^a^ Subjective opioid withdrawal symptoms

Figure 1 clarifies the relationship between the recorded total SOWS scores and the measured serum methadone concentrations, illustrating a weak correlation with wider confidence intervals at lower and higher concentrations. In the unadjusted LMM analysis (Table 2), we found statistically significant inverse associations, although weak to moderate correlations, between serum methadone concentrations and total SOWS scores (*p* = 0.011), and for the specific symptoms of anxiety (*p* = 0.009), bone and muscle aches (*p* = 0.007), and restlessness (*p* = 0.021) out of the 16 subjective opioid withdrawal symptoms based on the SOWS questionnaire, as well as for use of heroin (*p* = 0.028) and alcohol (*p* = 0.008). Except for a significant direct association with nausea reported as an adverse effect of methadone (*p* = 0.040), no associations were found between the other subjective adverse medication effects and methadone serum concentrations.

**Table 2:** The unadjusted associations between serum methadone concentrations and the study variables in 83 participants. All the primary and secondary outcome variables were inserted one by one in linear mixed model

	β-coefficient	95% CI^a^	*p*-value
Total SOWS^b^ score	− 3.9	− 7, − 9.1	0.011
Anxiety	− 4.3	− 7.6, − 1.1	0.009
Yawning	− 0.5	− 3.5, 2.5	0.739
Perspiring	− 0.4	− 4.1, 3.3	0.821
Tearing	− 1.5	− 4.1, 1.2	0.283
Nose run	− 1.7	− 4.9, 1.5	0.305
Goose bumps	− 2.1	− 5.5, 1.4	0.244
Shaking	− 2.7	− 5.7, 0.3	0.081
Hot flushes	− 2.6	− 6.2, 8.9	0.143
Cold flushes	− 3.2	− 7.1, 0.8	0.115
Bone- and muscle ache	− 4.9	− 8.4, − 1.4	0.007
Restlessness	− 5.2	− 9.6, − 0.8	0.021
Nausea	− 1.5	− 4.2, 1.2	0.272
Vomiting^d^	–	–	–
Muscle twitches	− 0.9	− 4.2, 2.4	0.590
Stomach cramps	− 1.5	− 4.2, 1.1	0.263
Feel like using^d^	–	–	–
Subjective adverse effects^d^	–	–	–
Euphoria^d^	–	–	–
Perspiring (as adverse effect)	1.4	− 3.0, 5.9	0.526
Nausea (as adverse effect)	3.1	0.1, 6.1	0.040
Concentration difficulties	− 0.7	− 4.7, 3.3	0.731
Feeling “brain fog”	1.5	− 2.2, 5.2	0.427
Decreased sexual desire	2.2	− 2.4, 6.8	0.351
Self-reported substance use ^c^	0.2	− 0.9, 1.3	0.754
Heroin	− 2	− 3.8, − 0.2	0.028
Other opioids^d^	–	–	–
Benzodiazepines	0.6	− 3.7, 4.9	0.772
Cannabis	4.7	− 0.5, 9.9	0.077
Amphetamines	− 0	− 3.0, 2.9	0.984
Alcohol	− 4.5	− 7.8, − 1.2	0.008

^a^ Confidence interval

^b^ Subjective opioid withdrawal symptoms

^c^ Frequent use (from daily to several times a month) of illicit drugs and alcohol during the month prior to surveys

^d^ The linear mixed model system was not able to analyze these variables due to scant data

### Adjustment of the results for confounding factors

When adjusting in the LMM analyses for age, gender, and the absolute time difference between blood sampling and the recording of the symptoms (Table 3), we found that the associations between serum methadone concentrations and total SOWS scores; the specific withdrawal symptoms of anxiety, bone and muscle aches, and restlessness; and use of heroin and alcohol still remained highly significant. There was a tendency toward higher serum concentrations among those who reported nausea as an adverse effect (*p* = 0.057). Obtaining *p*-values of < 0.10 by analyzing the withdrawal symptom of shaking as well as use of cannabis in the unadjusted LMM, we also added these variables to the adjusted model and found slight associations with serum methadone concentrations (*p* = 0.046 and *p* = 0.049, respectively).

**Table 3:** Associations between serum methadone concentrations and the study variables in 83 participants by using adjusted^a^ linear mixed model

	β-coefficient	95% CI ^b^	*p*-value
Total SOWS^c^ Score	− 4.3	− 5.6, − 2.9	< 0.001
Anxiety	− 0.5	− 0.8, − 0.2	0.004
Bone- and muscle ache	− 0.5	− 0.9, − 0.2	0.003
Restlessness	− 0.5	− 9.7, − 0.9	0.017
Shaking	− 0.3	− 0.6, − 0	0.046
Nausea (as adverse effect)	0.3	− 0.1, 0.6	0.057
Heroin use	− 0.2	− 0.4, − 0	0.015
Alcohol use	− 0.4	− 0.7, − 0.1	0.011
Cannabis use	0.5	0, 10.4	0.049

^a^ Adjusted for age, gender and absolute time difference between blood sampling and record of the symptoms

^b^ Confidence interval

^c^ Subjective opioid withdrawal symptoms

## Discussion

In clinical practice, there are different perceptions regarding what methadone dose adjustments should be based on: subjective symptoms or serum concentration measurements, and evidence on this topic is scarce. In this naturalistic cohort study, we have shown associations between serum methadone concentrations and subjective opioid withdrawal symptoms. Previous research has reported an inverse relationship between serum methadone concentrations and general objective withdrawal symptoms [20, 21, 24], but to our knowledge, no studies have investigated such relationships with subjective withdrawal symptoms. Although the correlations with the serum methadone concentration were not very strong for these symptoms, the findings are in line with existing theoretical expectations and support dose adjustments based on patient-reported symptoms in clinical practice.

A lower serum concentration-to-dose ratio among more than half the study participants who experienced withdrawal symptoms is a remarkable finding, meaning that serum concentrations were lower in this group despite these participants’ having methadone doses comparable to those of participants who did not report such symptoms. This outcome may be partially explained by the fact that increasing the dose was not met by a corresponding increase in serum methadone concentration. Considering possible aberrant methadone metabolisms or the influence of other disturbing factors, individualized dose optimization based on appropriate risk–benefit assessments might be emphasized as an approach capable of achieving treatment effects [5–8, 12, 29]. Among those not experiencing the optimal effect despite increasing the dose, dividing the daily dose or converting to another opioid such as long-action morphine can be considered [11, 24, 30]. When none of these measures can help, other causes such as pharmacodynamic factors and genetic variations affecting opioid receptors might be excluded [6, 31]. Finally, diversion of prescribed methadone take-home doses may be considered as an explanation for lower serum concentrations in the present study despite patients’ receiving appropriate doses and frequently being observed while taking their medications. Measurements of serum concentrations can be considered in such aberrant cases as a support to clinical decisions.

The higher use of heroin among those with lower serum methadone concentrations is also in line with earlier studies [20, 21, 23], indicating that even heroin use may prompt physicians to consider dose escalation. Using higher methadone doses is more effective in reducing heroin use and improving treatment retention [32–34]. However, some patients continue using heroin for its euphoric effects or other reasons, regardless of their serum methadone concentration. In addition, it is not clear whether the lower serum concentration causes the heroin use or whether some patients intentionally do not use the full dose prescribed to allow the heroin to be felt. It is challenging to answer these questions considering the naturalistic design of the study. Our finding of higher alcohol use among those with lower serum methadone concentrations could be explained by self-experienced replacement to alleviate opioid withdrawal symptoms. Research has also demonstrated an overlapping effect of alcohol on mu-opioid receptors in the central nervous system [35]. In addition, regular low-dose alcohol intake (< 4 alcoholic drink/day) may induce P450 enzymes and thus decrease serum methadone concentrations [26]. Considering the increased risk of adverse effects and overdose with the concurrent use of opioids and alcohol, a balanced dosage strategy is important to increase treatment retention and avoid not only relapse but also toxicity.

Although all participants reported at least one subjective adverse effect related to methadone treatment, none of these symptoms were significantly related to serum concentrations, except for a slight association with nausea. Studies on such associations are lacking. Nevertheless, it is important to keep in mind other possible physical and psychosocial conditions surrounding the patient, which may influence the total subjective experience and satisfaction with the treatment [10]. The inability to identify some associations in the present study may also be due to the group-level investigation. Following individuals over time with different doses may have revealed some associations. Additionally, we used self-reported responses to a locally developed questionnaire on common subjective adverse effects, not a validated instrument, which could have more accurately captured possible adverse effects.

The participants with higher serum methadone concentrations seemed to use more cannabis. Although the slight association may be due to a type-I error, the finding is of theoretical and clinical interest. A possible mechanism may be a central-acting effect to counterbalance undesirable side effects related to methadone treatment, for instance, an antiemetic effect of cannabis [36]. Some researchers have also suggested an opioid-saving effect of cannabis in patients with opioid dependence [37]. However, use of cannabis was not associated with MMT retention in a review article [38] and could not predict treatment outcomes such as relapse to heroin use or psychosocial functioning. To our knowledge, clinical studies have not yet examined possible associations between cannabis use and the methadone dose or serum concentration.

### Strengths and limitations

#### Strengths

The prospective nature of our study and the treatment platform allowed us to continuously meet the patients or repeat records if needed. We were thus able to manage the data collection more closely and reduce information bias. This design might be considered as a strength of the study.

#### Limitations

We were not able to perform all the interviews and blood tests at the same time (delayed follow-up), as some patients did not attend the planned interviews or could not complete the measurements at the same time. As a result, approximately half of those who had completed the primary surveys were eligible to be included in the study. Furthermore, although the study found associations between some subjective symptoms and serum methadone concentrations, the results must be interpreted in light of the relatively small effect sizes. The observed weak to moderate correlations may reflect possible influences of other factors such as concurrent use of illicit drugs or abstinence from these substances, comorbid somatic and psychiatric conditions, or even manipulation of symptoms to receive higher methadone doses. Another limitation may be the small sample size and possibility for some uncovered associations. Further clinical studies are needed to obtain more knowledge in this field.

## Conclusions

Subjective opioid withdrawal symptoms – particularly anxiety, bodily pain, restlessness, and shaking (slightly) – and self-reported use of heroin and alcohol were associated with lower serum methadone concentrations. Patients with higher serum methadone concentrations had a tendency to use more cannabis. As the concentration of methadone in serum was related to the patient’s self-reported symptoms and use of substances, such symptoms may support the need for dose adjustments. Dividing the dose or converting to other opioids should be considered when dose escalations do not relieve the symptoms. Measurements of serum concentrations can be considered in some aberrant cases as a support to clinical decisions.

## Data Availability

All data generated or analyzed during this study are included in this manuscript. No additional data are available due to data protection requirements.

## Abbreviations

CDR: Concentration-to-dose ratio
CI: Confidence interval
DOT: Direct observed treatment
LMM: Linear mixed model
MMT: Methadone maintenance treatment
OAT: Opioid agonist therapy
SOWS: Subjective opioid withdrawal scale
TDM: Therapeutic drug monitoring
